# Supplementary material for: RIM1α SUMOylation Is Required for Fast Synaptic Vesicle Exocytosis
Source: Cell Rep. 2013 Nov 27;5(5):1294–301. doi: 10.1016/j.celrep.2013.10.039 (PMC3898736; doi:10.1016/j.celrep.2013.10.039)
Supplement: Document S1. Supplemental Experimental Procedures and Figures S1–S3 [file mmc1.pdf]

Supplemental Experimental Procedures

Molecular Biology: Cloning of all constructs was carried out using standard molecular biology methods. For SypHy and SyGCaMP3 experiments, SypHy or SyGCaMP3 were expressed on a pFIV plasmid which also expressed RIM1 shRNA (vector made in house, shRNA target sequence **gaggaggagaggaacattat** of *Rattus Norvegicus* RIM1α). RIM1α rescue constructs (carrying silent point mutations which made RIM1α insensitive to shRNA) were expressed either in a custom made mCherry-IRES-RIM1α vector or modified from pCMV-HA-RIM1α. FM dye experiments used a pFIV vector expressing RIM1 shRNA and mCherry. For all knockdown studies, 'Control' cells were transfected with pFIV (expressing mCherry, SypHy or SyGCaMP3) without shRNA. GST-tagged $Ca_V2.1$ PDZ ligand was produced by cloning synthetic DNA oligonucleotides into pGEX4T-1 vector. All subcloning and mutagenesis reactions were performed according to standard protocols. Sindbis viruses were produced from pSinRep5 vector as described previously (Craig et al., 2012).

Biochemistry: For all immunoprecipitations, antibodies were bound to Protein G sepharose. SUMO co-immunoprecipitation was performed from cultured rat cortical neurones (Martin et al., 2007) (DIV 18) using 1 µg of anti SUMO D11 antibody (Santa Cruz). Cells were lysed on ice (lysis buffer: 150 mM NaCl, 25 mM HEPES, 1% Triton and 0.1% SDS – pH 7.4) and incubated at 37°C for 30 minutes with or without 20 nM NEM before immunoprecipitation. Western blotting was performed using anti-RIM1/2 antibody (Synaptic Systems). For the HEK293T SUMOylation assay (Craig et al., 2012), cells were transfected with RIM1α-HA, Ubc9-Flag and either active SUMO-1-GG-YFP or non-conjugatable SUMO-ΔGG-YFP for 48 hours before lysis on ice in buffer containing 20 nM NEM. HA co-immunoprecipitation was performed using 2 µg mouse anti-HA antibody (Sigma) and anti-GFP (Roche). For interactor studies, RIM1α co-immunoprecipitation was performed from neurones infected with Sindbis virus encoding WT or K502R RIM1α-HA for 18 hours using 2 µg of mouse/rabbit anti-HA antibody (Sigma). For Rab3 interactions, immunoprecipitations were carried out in the presence of 0.5 mM GTPγS. Samples were lysed on ice in buffer with or without 20 nM SENP1 and blotted for the protein of interest. Free glutathione-S-transferase (GST) and the $Ca_V2.1$ PDZ ligand plus two preceding amino acids (sequence SEDDWC) fused to GST were expressed in BL21 (DE3) *E.Coli* and purified on glutathione sepharose beads. Affinity pulldowns were performed from neuronal lysate infected with WT or K502R RIM1α-HA (lysis/binding buffer: 300 mM NaCl, 25 mM HEPES, 2 mM DTT and 1% Triton – pH 7.4. Wash buffer in mM: 150 mM NaCl, 25 mM HEPES, 1 mM DTT and 1% Triton – pH 7.4).

Antibodies: Other antibodies used were anti-RIM1 (BD Biosciences), anti-βIII tubulin (Sigma Aldrich), mouse/rabbit anti-HA (Sigma Aldrich), anti-GST (Amersham), anti-GFP (Roche), mouse anti-Synapsin1 (BD Biosciences), rabbit anti-Synapsin1 (Novus Biologicals), anti-Munc13 (BD Biosciences), anti-Rab3 (BD Biosciences), anti-Liprin α3 (Synaptic Systems), anti-ERC1b/2 (ELKS1b/2) (Synaptic Systems), anti-$Ca_V2.1$ (Alomone Labs) and anti-SUMO-1 21C7 (Developmental Studies Hybridoma Bank, Iowa).

Immunocytochemistry: Immunocytochemistry assays were performed with paraformaldehyde fixation according to standard protocols. Cells were permeabilized in 0.1% Triton except for SUMO-1 staining, in which 20 µg/ml digitonin was used in order to avoid nuclear permeabilisation and thereby reduce the intense SUMO-1 staining normally seen in cell nuclei. All quantification was performed using ImageJ software. In all cases, n = mean of 10-12 ROIs taken from one cell. At least three independent experiments (i.e. on different neuronal cultures on different days) were performed in all cases, with all results normalised to the average of the control values (set to 100%) for each batch of cells. For colocalisation experiments, Mander's test of colocalisation was used. For Fig. 1D, the Mander's M1 value recorded represents the overlap of SUMO-1 signal with that of RIM1/2. For Supplemental data Fig. 1E-F, the Mander's M1 value represents the overlap of HA (RIM1α) signal with that of Synapsin-1 and values are normalised to the mean WT RIM1α colocalisation index. For $Ca_V2.1$ clustering experiments, synaptic $Ca_V2.1$ was identified by picking ROIs from Synapsin-1 dense regions (as in (Kaeser et al., 2011)).

Cell Culture: Embryonic cortical and hippocampal neurones were isolated and maintained as previously described (Martin and Henley, 2004). For all biochemistry, cortical neurones were used at DIV18. For all live cell imaging, hippocampal neurones were used at DIV15. For FM-dye experiments, hippocampal neurons were typically transfected at DIV 11 with RIM1 shRNA (mCherry) and either WT or non-SUMOylatable (K502R) RIM1α-HA rescue and imaging experiments were performed four days later. For SypHy and SyGCaMP3 experiments, transfections were performed in a similar manner, but with SypHy or SyGCaMP3 expression on the shRNA vector and a mCherry-IRES-RIM1α rescue construct. All neuronal transfections were performed using Lipofectamine 2000 (Invitrogen) according to manufacturer's instructions.

FM1-43 Experiments: FM1-43 dye loading and unloading was carried out as described (Gaffield and Betz, 2006). Cells were kept in HBS (in mM: 140 NaCl, 5 KCl, 1.8 $CaCl_2$, 0.8 $MgCl_2$, 25 HEPES and 0.9 g/litre glucose - pH 7.4) with 25 μM CNQX and 50 μM D-AP5 throughout. The entire releasable pool of vesicles was FM labelled by stimulating with 45 mM KCl for 90 seconds in the presence of dye, followed by an additional 90 seconds of dye exposure. Following 10 minutes of washing in dye-free solution, cells were imaged. Images of FM1-43 fluorescence were taken every 5 seconds by confocal microscopy. Baseline fluorescence was captured for 60 seconds before a stimulation of 600 action potentials (APs) at 20 Hz was applied to induce bulk exocytosis and thus dye unloading. After 2 minutes, the nerve terminal was stimulated with 900 APs at 30 Hz to enable complete unloading of all releasable FM dye (to reveal terminal background). For each cell, 12 ROIs were analysed where each ROI encapsulated a single synaptic bouton. Values were normalised to terminal background. Slope analysis was performed on the initial 15 seconds of stimulation (during which all profiles were linear) by linear regression.

Synaptophysin-pHluorin (SypHy) Experiments: SypHy experiments were performed as described (Burrone et al., 2006). Exocytosis was triggered using electrical field stimulation, using 1 ms pulses (APs) of either 20 mA or 50 V (which were found to yield identical results). RRP stimulation was triggered by 40 APs at 20 Hz. Fusion of all releasable synaptic vesicles (reserve pool or RP)_was triggered by 600 APs at 20 Hz. All experiments were performed in HBS with 50 μM D-AP5, 25 μM CNQX and 1 μM bafilomycin A. Perfusion with NH_4Cl (pH 7.4), was used to reveal total levels of SypHy loading of neurones at the end of each experiment. Images were taken at 0.5,2 or 10 Hz depending on the experiment, using a CCD camera with a GFP filter cube. For each cell, 13 ROIs were analysed, with non-responsive ROIs discounted. Data were first normalised to background levels (i.e. $\Delta F/F_0$), and then expressed as a percentage of fluorescence after NH_4Cl perfusion (F_{max}). Slope analysis was performed by linear regression on the initial 10 seconds of stimulation (during which all profiles were linear) for RP release, and for the full 2 seconds of stimulation for RRP release.

SyGCaMP3 Experiments: All experiments were performed in HBS with 25 μM CNQX and 50 μM D-AP5. As with SyPhy experiments, RRP stimulation was triggered by 40 APs at 20 Hz after steady baseline recordings were obtained. Images were taken at 10 Hz using a CCD camera with a GFP filter cube. Maximal SyGCaMP3 signal was obtained by 5 minute incubation with 5 μM ionomycin made up in HBS. Data were first normalised to background levels (i.e. $\Delta F/F_0$) and then expressed as a percentage of fluorescence after ionomycin perfusion (F_{max}). For all functional assays, data is collated from at least 3 individual experiments where n=one cell and for each cell, 10-13 ROIs were analysed. Image analysis was performed using ImageJ software as described (Gaffield and Betz, 2006).

Statistical Analysis: Graphpad Prism software (Graphpad Inc.) was used for graph plotting and statistical analysis. For comparison of multiple data sets, 1-way ANOVA with Bonferroni's post-hoc test was used. For comparison of 2 data sets, a 1-tailed Student's *t*-test was used. Curve fitting for Fig. 3C was performed using Graphpad Prism. Control and WT rescue traces were fitted with a Boltzman Sigmoidal curve whereas KD and K502R rescue traces were fitted with linear regression. All data is reported as mean ± SEM.

Supplemental Figure Titles and Legends

Figure S1 – A501S RIM1α is non-SUMOylatable, **Related to Figure 1**

A: Confirmation that lysine 502 is the only SUMOylation site on RIM1α. WT and A501S RIM1α were used in the SUMOylation assay in HEK293T cells (Fig. 1F,G). Western blot for HA (RIM1α).

Figure S2– RIM1 knockdown-rescue, equal expression and normal targeting/interaction of K502R RIM1α, and FM1-42 uptake in KD-A501S rescue cells, **Related to Figures 2, 3 and 4.**

A: Representative blot showing knockdown of exogenously expressed RIM1α-HA and rescue with both WT and non-SUMOylatable K502R RIM1α in HEK293T cells. β-tubulin is shown as a loading control.
B: Quantification of (A). RIM1α expression levels are expressed as % control cells not transfected with RIM1 shRNA. (n=4) ***p<0.001 (1-way ANOVA). Data are represented as mean ± SEM.
C: Representative images for hippocampal neurones stained for RIM1/2 (green) after cotransfection with RIM1 shRNA (mCherry, red) and either WT or K502R RIM1α rescue (green). Scale bar - 10 µm.
D: Quantification of (C). RIM1α expression levels are expressed as % control cells not transfected with RIM1 shRNA. (n=10-16) ***p<0.001 (1-way ANOVA). Data are represented as mean ± SEM.
E: Representative images for RIM1α-HA colocalisation with Synapsin-1. Hippocampal neurones infected with Sindbis virus expressing either WT or K502R RIM1α-HA stained 18 hours after infection for presynaptic protein Synapsin-1 (red) and HA (green). Arrows highlight regions of colocalisation. Scale bar - 5 µm.
F: Quantification of (E). Mander's M1 coefficient for colocalisation of HA (RIM1α) with Synapsin-1 (n=6, 6 ROIs per cell). Data are expressed as % of the M1 coefficient of the WT condition. Data are represented as mean ± SEM.
G: Representative images for FM1-43 dye uptake by high K^+ (45 mM) stimulation in hippocampal neurones. Scale bar - 5 µm.
H: Quantification of (B). FM1-43 uptake for control, RIM1 KD, WT rescue, K502R rescue and A501S rescue conditions. Normalised fluorescence refers to average FM1-43 pixel intensity as % mean fluorescence of control neurones. (n=5-8) ***p<0.001 (1-way ANOVA). Data are represented as mean ± SEM.

Figure S3 – RIM1α interaction with Liprin α3 and Rab3 is not SUMO-dependent and 3KN RIM1α does not bind $Ca_V2.1$, **Related to Figure 4.**

A: Representative Rab3, Liprin α3 and ELKS1b/2 blots of HA (RIM1α) immunoprecipitation from cortical neurones virally infected with WT or K502R RIM1α-HA.
B: Quantification of (A) (n=3-4). Data are represented as mean ± SEM.
C: Representative blots showing GST-$Ca_V2.1$-PDZ interaction with WT and 3KN RIM1α in HEK293T cells. Pulldowns with GST-$Ca_V2.1$-PDZ were blotted for HA (RIM1α) and GST.
D: Quantification of (C) (n=3). Data are presented as % WT interaction. ***p<0.001 (1 way ANOVA). Data are represented as mean ± SEM.

A

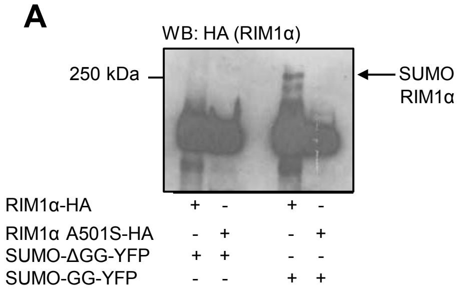

Girach et al. – **Figure S1**

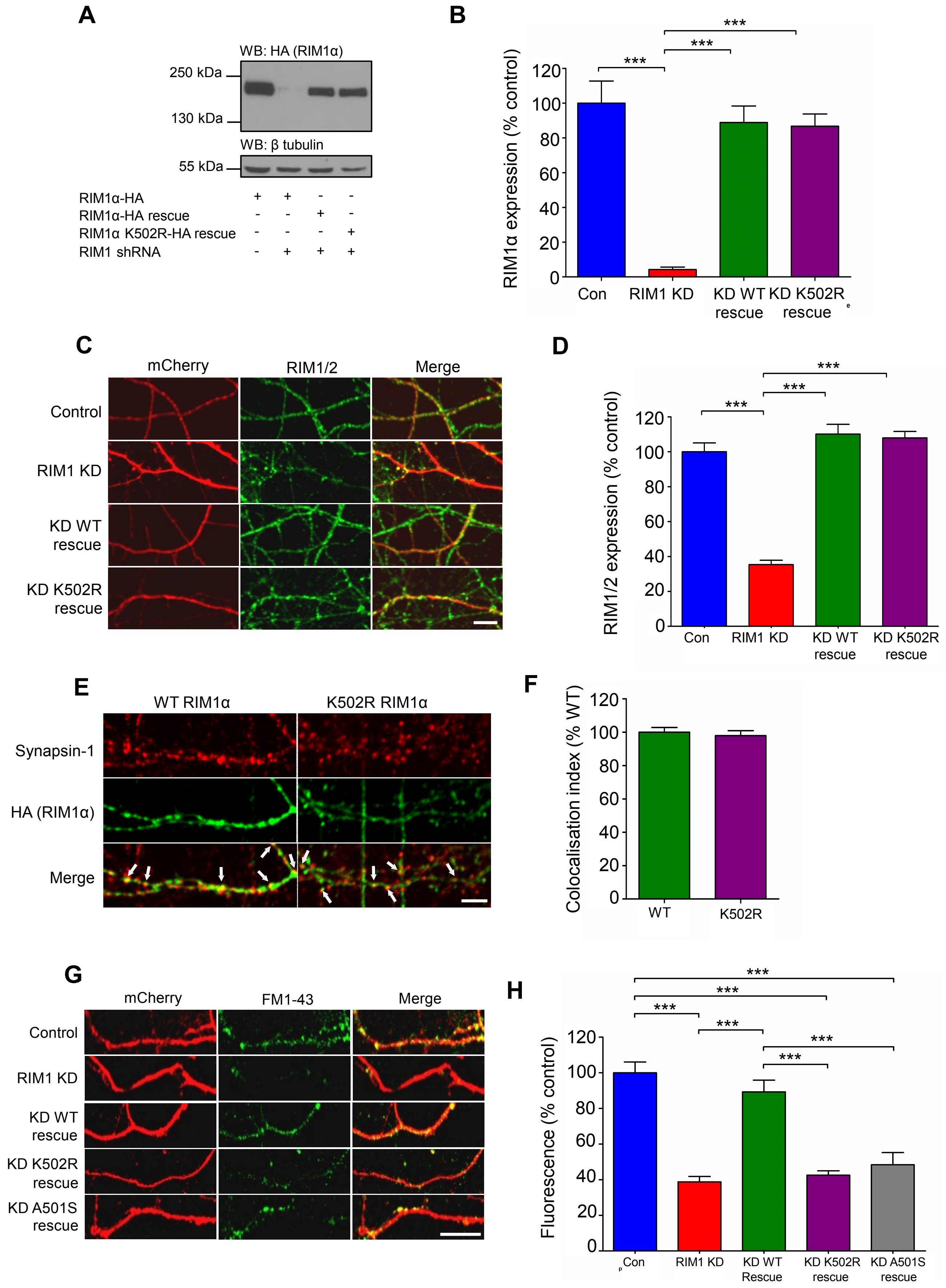

Girach et al. – **Figure S2**

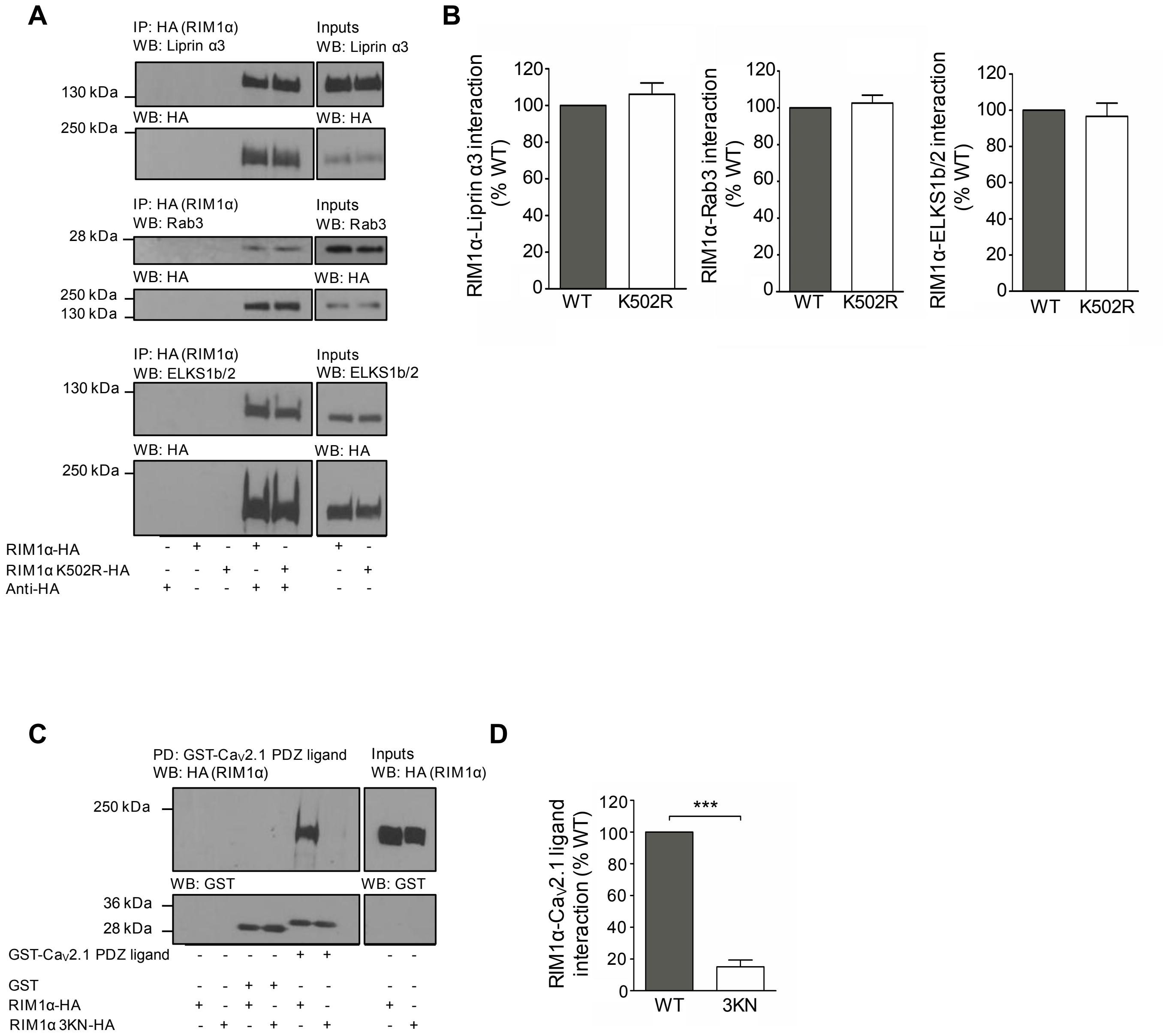

Girach et al. – **Figure S3**
