# Supplementary material for: RIM1α SUMOylation Is Required for Fast Synaptic Vesicle Exocytosis
Source: Cell Rep. 2013 Nov 27;5(5):1294–301. doi: 10.1016/j.celrep.2013.10.039 (PMC3898736; doi:10.1016/j.celrep.2013.10.039)
Supplement: Document S2. Article plus Supplemental Information [file mmc2.pdf]

Cell PRESS
Open ACCESS

Cell Reports
Article

RIM1α SUMOylation Is Required for Fast Synaptic Vesicle Exocytosis

Fatima Girach,[1,2] Tim J. Craig,[1,2] Daniel L. Rocca,[1] and Jeremy M. Henley[1,*]
[1]Department of Biochemistry, School of Medical Sciences, University Walk, University of Bristol, Bristol BS8 1TD, UK
[2]These authors contributed equally to this work
*Correspondence: anjmh@bristol.ac.uk
http://dx.doi.org/10.1016/j.celrep.2013.10.039
This is an open-access article distributed under the terms of the Creative Commons Attribution License, which permits unrestricted use, distribution, and reproduction in any medium, provided the original author and source are credited.

SUMMARY

The rapid, activity-dependent quantal presynaptic release of neurotransmitter is vital for brain function. The complex process of vesicle priming, fusion, and retrieval is very precisely controlled and requires the spatiotemporal coordination of multiple protein-protein interactions. Here, we show that posttranslational modification of the active zone protein Rab3-interacting molecule 1α (RIM1α) by the small ubiquitin-like modifier 1 (SUMO-1) functions as a molecular switch to direct these interactions and is essential for fast synaptic vesicle exocytosis. RIM1α SUMOylation at lysine residue K502 facilitates the clustering of $Ca_V2.1$ calcium channels and enhances the Ca^{2+} influx necessary for vesicular release, whereas non-SUMOylated RIM1α participates in the docking/priming of synaptic vesicles and maintenance of active zone structure. These results demonstrate that SUMOylation of RIM1α is a key determinant of rapid, synchronous neurotransmitter release, and the SUMO-mediated "switching" of RIM1α between binding proteins provides insight into the mechanisms underpinning synaptic function and dysfunction.

INTRODUCTION

Activity-dependent neurotransmitter release is mediated by the Ca^{2+}-dependent fusion of synaptic vesicles at the active zone of the presynaptic membrane (Südhof and Rizo, 2011). Rab3-interacting molecule 1α (RIM1α) interacts, either directly or indirectly, with most active zone proteins (Calakos et al., 2004) and is crucial to active zone function (Wang et al., 1997). More specifically, it participates in vesicle priming via interactions with Munc13-1 (Deng et al., 2011; Koushika et al., 2001), Ca^{2+} channel clustering near release sites (Coppola et al., 2001; Kaeser et al., 2011), and synaptic plasticity, including presynaptic LTP (Castillo et al., 2002) and homeostatic plasticity (Müller et al., 2012). Furthermore, interactions between RIM1α and Rab3a (Lonart, 2002; Wang et al., 1997) and synaptotagmin (Coppola et al., 2001) suggest roles in vesicle docking and Ca^{2+} triggering of exocytosis, respectively. Thus, RIM1α acts as a hub in a diverse range of functions, but it is unknown how RIM1α binding to its multiple interacting proteins is regulated.

Posttranslational protein modification by SUMOylation is a fundamentally important regulatory mechanism in nearly all cell pathways (Hay, 2005). Small ubiquitin-like modifier 1 (SUMO-1) is a 97-residue peptide that attaches to proteins via an isopeptide bond to the primary amine groups of lysine residues. This covalent attachment is catalyzed by the E2 enzyme Ubc9, which binds to the substrate protein, and is removed by SUMO-specific proteases (SENPs) (Flotho and Melchior, 2013). In neurons, SUMOylation participates in the regulation of synapse formation (Shalizi et al., 2006), neurotransmitter receptor trafficking, synaptic plasticity (Martin et al., 2007; Craig et al., 2012; Jaafari et al., 2013), and presynaptic neurotransmitter release (Feligioni et al., 2009). However, most of the SUMO substrate proteins mediating these effects are unknown.

In this study, we identify RIM1α as a synaptic SUMO substrate. Abrogation of RIM1α SUMOylation leads to severe defects in action potential (AP)-evoked presynaptic exocytosis and Ca^{2+} entry, but not vesicle docking or priming. We show that inhibition of RIM1α SUMOylation dramatically reduces its PDZ domain interaction with $Ca_V2.1$ and suggest that RIM1α SUMOylation serves to delineate the many different functions of this protein.

RESULTS AND DISCUSSION

RIM1 Is a Neuronal SUMO Substrate

To identify neuronal SUMOylation substrates, we used GST-tagged Ubc9 to affinity purify binding proteins from extracts of rat cortical neurons. Mass spectrometry and western blotting showed that RIM interacts with Ubc9 (Figure 1A). Anti-SUMO-1 antibody immunoprecipitated a RIM1/RIM2-reactive band of the correct predicted molecular weight, which was protected by NEM, which inhibits SENP-mediated deSUMOylation (Figures 1B and 1C). Consistent with RIM being a SUMO substrate, RIM1/RIM2 and SUMO-1 show extensive colocalization (Mander's M1 colocalization coefficient of 0.6870 ± 0.01, where M1 represents the amount of SUMO-1 fluorescence that overlaps RIM1/RIM2 fluorescence, n = 35) in the processes of hippocampal neurons (Figure 1D). This colocalization shows that SUMO-1

CrossMark

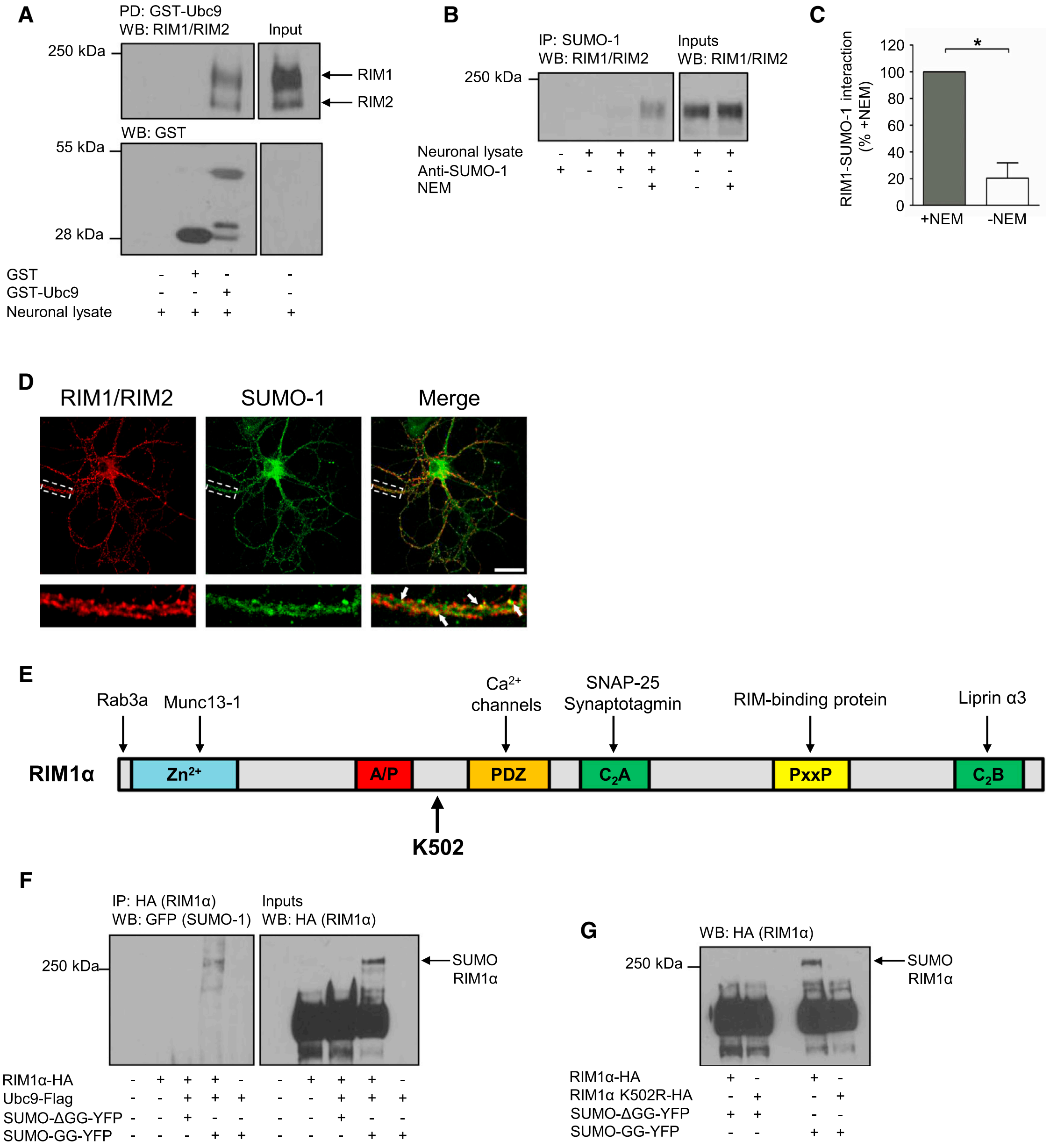

Figure 1. RIM1 Is a SUMO Substrate in Neurons

(A) GST-Ubc9 pull-down (PD) for RIM1/RIM2 from rat cortical lysate is shown. WB, western blot.

(B) Coimmunoprecipitation of RIM1/RIM2 with SUMO-1 from cortical neurons is presented. IP, immunoprecipitation.

(C) Quantification of (B) is expressed as the percent (%) RIM1/RIM2 immunoprecipitated in the presence of NEM (n = 3). *$p < 0.05$ (Student's t test). Data are represented as mean ± SEM.

(D) Representative images present RIM1/RIM2 (red) and SUMO-1 (green) immunostaining in hippocampal neurons. Panels below show magnification of the area in the dashed boxes. Arrows highlight colocalization. The scale bar represents 20 μm.

(E) Schematic shows significant features and binding sites in RIM1α.

(F) Coimmunoprecipitation of SUMOylated RIM1α in HEK293T cells is presented. Samples immunoprecipitated with HA (RIM1α) were blotted for GFP (SUMO). Representative of four blots is shown. SUMO-ΔGG is a nonconjugatable form of SUMO used as a negative control (cf. conjugatable SUMO-GG).

(G) Western blot shows that K502R mutation abolishes RIM1α SUMOylation in HEK293T cells.

See also Figure S1.

is present in the presynapse and thus has the potential to influence the presynaptic functions of RIM1/RIM2, although it is likely that there are many presynaptic substrates.

In subsequent experiments, we focused on the RIM1α isoform because of the higher abundance and well-characterized presynaptic role (Figure 1A; Schoch et al., 2006). RIM1α is a

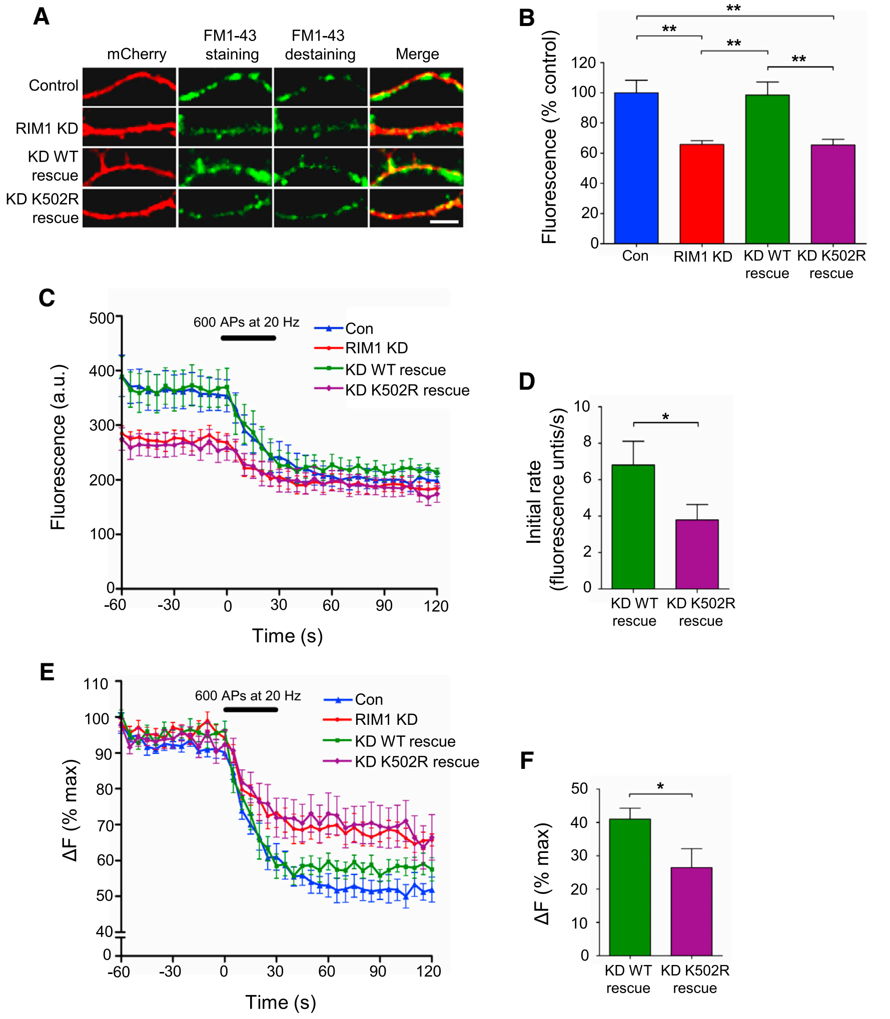

Figure 2. RIM1α SUMOylation Is Involved in Synaptic Vesicle Cycling

(A) Representative images show FM1-43 uptake in hippocampal neurons treated with shRNA RIM1 KD and rescue with WT or nonSUMOylatable K502R RIM1α. Destained images correspond to 30 s poststimulation. The scale bar represents 5 μm.

(B) Quantification of (A) is presented. Normalized fluorescence is FM1-43 pixel intensity as percentage (%) of mean fluorescence of control (Con) neurons (n = 9–13). **$p < 0.01$ (one-way ANOVA). Data are represented as mean ± SEM.

(C) Time course shows FM1-43 unloading triggered by 600 APs at 20 Hz for control, RIM1 KD, WT rescue, and K502R rescue neurons (n = 9–13), normalized to terminal background. Data are represented as mean ± SEM.

(D) Quantification of the slope during the initial 15 s of stimulation in (C) is shown. *$p < 0.05$ (Student's t test). Data are represented as mean ± SEM.

(E) Data in (C) are normalized to the average baseline (60 s prestimulus).

(F) Mean normalized FM1-43 release in (E) after 30 s of stimulation is shown. *$p < 0.05$ (Student's t test). Data are represented as mean ± SEM. (% max), percent maximum.

See also Figure S2.

multidomain protein but contains only one lysine (K502) within a consensus SUMOylation motif (Figure 1E). We were able to SUMOylate RIM1α in a HEK cell-based SUMO assay using SUMO-GG (in which the C-terminal diglycine conjugation motif has been exposed), but not SUMO-ΔGG (in which the conjugation motif has been deleted) (Figure 1F). Mutation of this lysine to arginine (K502R) or mutation of hydrophobic residue in the consensus site (A501S) completely prevented RIM1α SUMOylation, confirming that K502 is the sole SUMO-1 attachment site (Figures 1G and S1A).

RIM1α SUMOylation Regulates the Synaptic Vesicle Cycle

We used shRNA to knock down endogenous RIM1 and replaced it with nonSUMOylatable K502R RIM1α. In HEK293T cells, there was a >90% knockdown (KD) of cotransfected RIM1α, and this was effectively rescued by shRNA-insensitive constructs ("rescue"; Figures S2A and S2B). In hippocampal neurons, there was a ~65% KD of endogenous RIM (Figures S2C and S2D), with equivalent levels of replacement with WT or K502R RIM1α that both displayed similar synaptic colocalization with synapsin-1 (Figures S2E and S2F). These results indicate that SUMOylation is not required for RIM1α localization at the active zone.

To determine the roles of RIM1α in presynaptic exocytosis, we used styryl FM dye loading (Gaffield and Betz, 2006). In RIM1 KD neurons, FM1-43 dye loading in response to depolarization was significantly reduced. Replacement with WT, but not K502R or A501S RIM1α, rescued this defect (Figures 2A, 2B, S2G, and S2H), indicating that RIM1 removal slows or inhibits the synaptic vesicle cycle and that SUMOylation of the replacement RIM1α is required to rescue this defect.

We next investigated exocytosis by measuring FM1-43 unloading in response to electrical field stimulation of neurons (Burrone et al., 2006), using 600 APs at 20 Hz, which induces exocytosis of the releasable synaptic vesicle pool (Fernández-Alfonso and Ryan, 2004). Neurons rescued with WT RIM1α responded identically to control, non-shRNA-treated cells, whereas in contrast, K502R RIM1α failed to rescue the KD phenotype (Figure 2C). Cells rescued with K502R RIM1α displayed a significant reduction in the initial rate of FM1-43 unloading (taken as the initial 15 s during which all profiles were linear) compared to cells rescued with WT RIM1α (Figures 2C and 2D). Additionally, the total amount of FM1-43 unloading was significantly lower in cells rescued with K502R RIM1α (Figures 2E and 2F). Together, these data show that RIM1α SUMOylation is necessary for normal stimulus-evoked synaptic vesicle exocytosis.

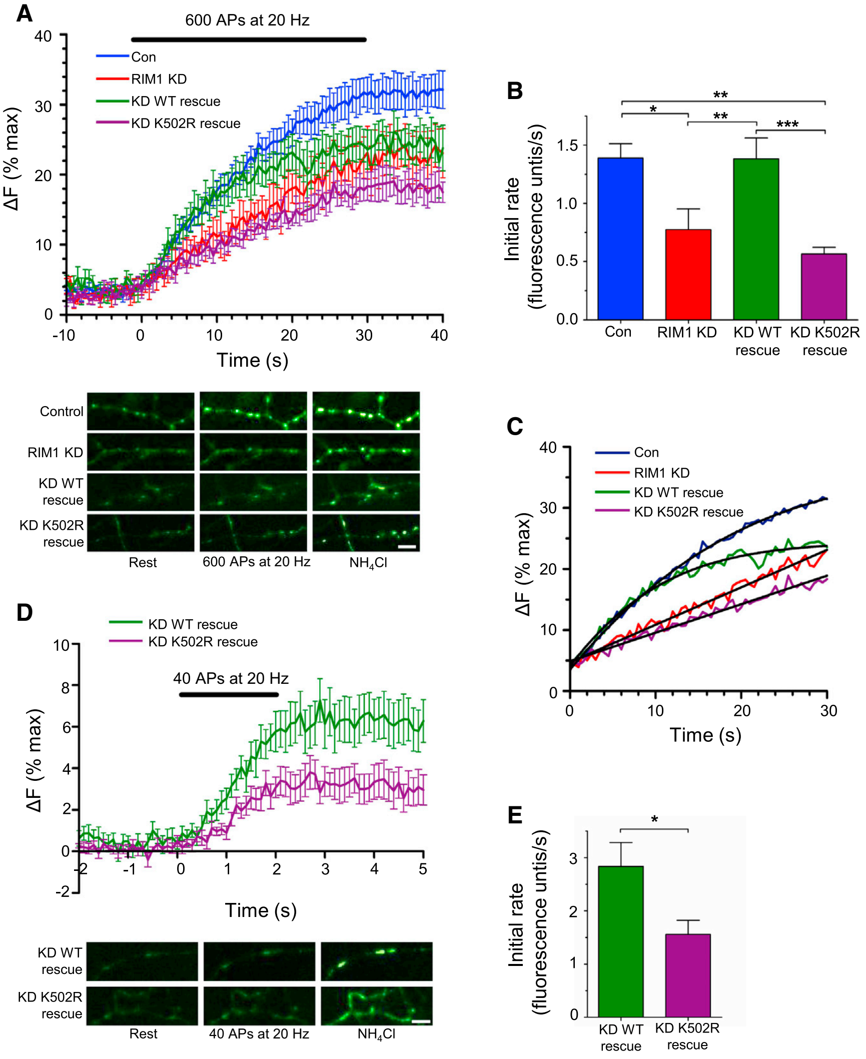

Figure 3. RIM1α SUMOylation Has a Critical Role in Fast Synaptic Vesicle Exocytosis

(A) SypHy fluorescence measurement of the releasable synaptic vesicle pool (n = 6–9) is shown. Fluorescence is normalized to baseline and expressed as percentage (%) of total SypHy signal. Panels are representative images of SypHy fluorescence taken at rest (0 s), after 600 APs at 20 Hz (30 s), and after NH_4Cl wash. Data are represented as mean ± SEM. The scale bar represents 5 μm.

(B) Quantification of the initial rate of exocytosis in (A) is presented. *$p < 0.05$, **$p < 0.01$, and ***$p < 0.001$ (one-way ANOVA). Data are represented as mean ± SEM.

(C) Data from (A) are plotted to show the 30 s period of field stimulation (error bars removed for clarity). Release profiles of control and WT rescue cells are best described by an exponential function, whereas RIM1 KD and K502R rescue cells follow a linear profile. Data are represented as mean.

(D) Exocytosis from RRP (n = 10), imaged at 10 Hz, is shown. Fluorescence is normalized to the baseline and expressed as percent (%) total SypHy signal (obtained with NH_4Cl). Panels below are representative images of SypHy fluorescence. Data are represented as mean ± SEM. The scale bar represents 5 μm.

(E) Quantification of the rate of RRP release (relative slope during the 2 s of stimulation) is presented. *$p < 0.05$ (Student's t test). Data are represented as mean ± SEM.

See also Figure S2.

RIM1α SUMOylation Has a Critical Role in the Fast Phase of Synaptic Vesicle Exocytosis, but Not Vesicle Docking or Priming

We further investigated the role of RIM1α SUMOylation using the fluorescent indicator synaptophysin-pHluorin (SypHy) to visualize vesicle fusion events (Burrone et al., 2006). Consistent with the FM1-43 experiments, RIM1 KD dramatically altered the kinetics of exocytosis of the synaptic vesicle pool (evoked by 600 APs at 20 Hz), and this was rescued by WT RIM1α, but not K502R RIM1α (Figures 3A–3C). The initial linear rate of exocytosis during the first 10 s of stimulation was reduced by ~50% in RIM1 KD and K502R RIM1α-rescued neurons compared to control and WT RIM1α-rescued neurons (Figures 3A–3C). Furthermore, as in the FM dye experiments, RIM1 KD neurons rescued with WT RIM1α displayed similar rates of release to controls not treated with shRNA, which was different from the rates observed in RIM1 KD and K502R RIM1α-rescued cells. However, although RIM1α WT expression rescues the initial vesicle release rate, it does not completely recover the total level of exocytosis (Figures 3A–3C). We attribute this to the KD of both RIM1α and RIM1β but rescue with only the RIM1α isoform. Thus, as previously reported by Kaeser et al. (2008), these results also implicate RIM1β in regulation of synaptic strength. In these experiments, the presence of bafilomycin A blocks vesicle reacidification, allowing specific measurement of exocytosis in the absence of endocytosis. Thus, we can state with confidence that the effects we see are specific for exocytosis, independent of any contribution of compensatory endocytosis.

Exocytosis from control and WT RIM1α-rescued cells displays a biphasic release profile, whereas the RIM1 KD and K502R RIM1α-rescued cells have a linear release profile (Figure 3C). To test whether this was due to the loss of the fast, initial phase of release from RIM1 KD and K502R-rescued neurons, we selectively measured primed synaptic vesicles ready to be released immediately on membrane depolarization (the readily releasable pool; RRP) using 40 APs at 20 Hz (de Jong et al., 2012) (Figure 3D). During the 2 s stimulation, there was an ~50% reduction in rate of exocytosis in cells rescued with K502R RIM1α compared with WT RIM1α-rescued cells (Figure 3E). These results show that exocytosis of the primed vesicles in the RRP is significantly impaired (resulting in a smaller apparent RRP) in the cells expressing K502R RIM1α. This defect in exocytosis is unlikely to be due to a defect in docking or priming of

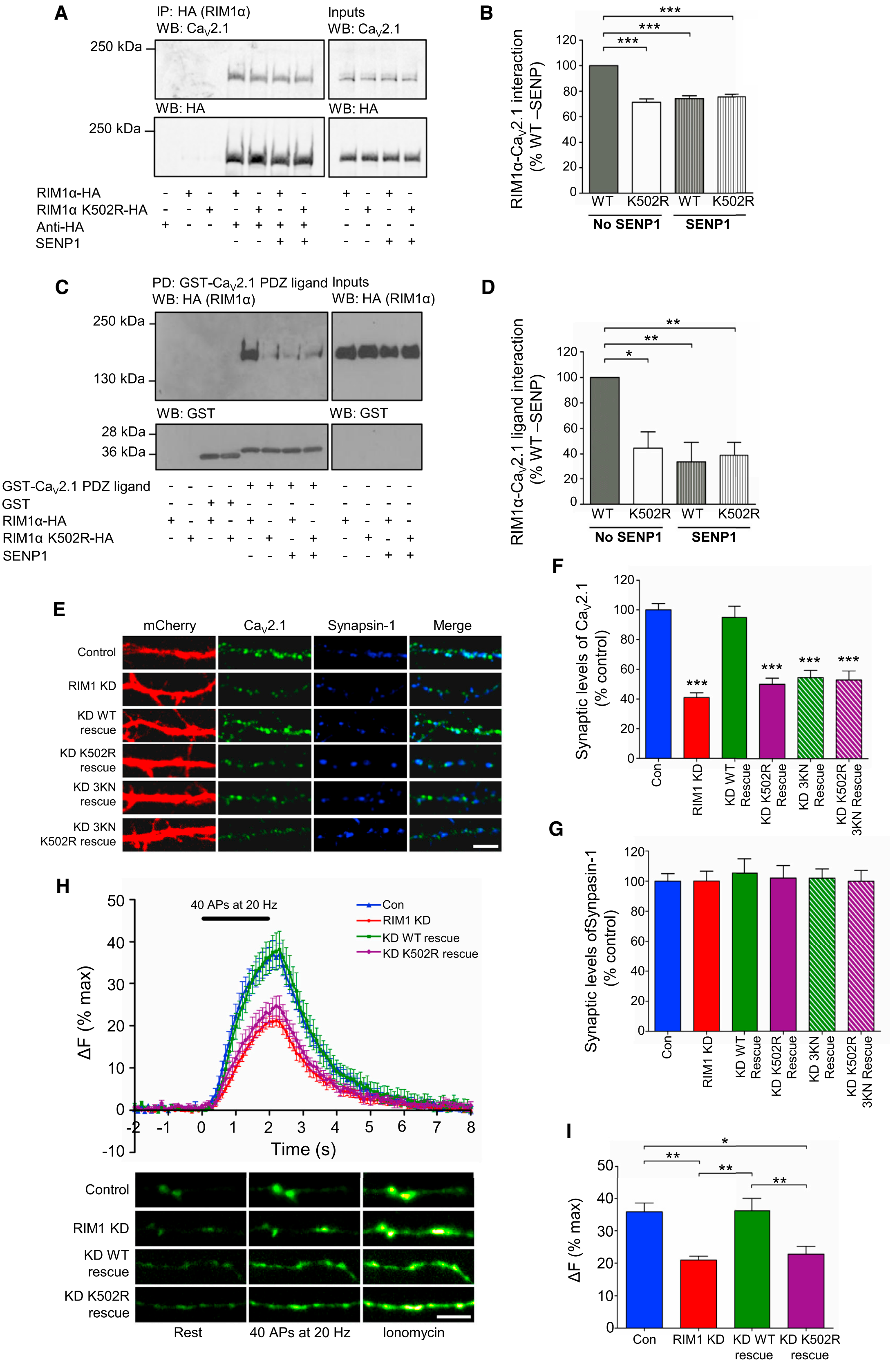

(legend on next page)

vesicles because this would result in a decrease in RRP size but the same rate of release. Thus, we conclude that RIM1α SUMOylation has a critical role in fast phase of synaptic vesicle exocytosis.

RIM1α SUMOylation Has a Critical Role in Depolarization-Evoked Presynaptic Ca^{2+} Entry due to a SUMO-Dependent PDZ Domain Interaction of RIM1α with $Ca_V2.1$

The initial fast phase of synaptic vesicle exocytosis is called "synchronous release" because it occurs in direct response to Ca^{2+} entry through Ca^{2+} channel clusters at release sites (Gundelfinger and Fejtova, 2012). RIM1α plays a major role in maintaining Ca^{2+} channel clusters via a direct PDZ interaction between RIM1α and P/Q-type Ca^{2+} channels (Kaeser et al., 2011) and through an indirect complex formation involving RIM-binding protein (Liu et al., 2011). Intriguingly, the altered kinetics of exocytosis we see are similar to previous reports using P/Q-type Ca^{2+} channel inhibitors (Li et al., 2011). Following expression in cortical neurons, nonSUMOylatable K502R RIM1α bound significantly less to $Ca_V2.1$ than WT RIM1α. Consistently, deSUMOylation of neuronal lysate using SENP1 reduced WT RIM1α binding to $Ca_V2.1$ to the same level as the K502R RIM1α mutant (Figures 4A and 4B). These results indicate that RIM1α SUMOylation directly regulates its binding to $Ca_V2.1$. Importantly, consistent with RIM1α active zone localization (Schoch et al., 2002) and vesicle docking (Dulubova et al., 2005) being unaffected by SUMOylation, WT and K502R RIM1α display equivalent binding to other active zone proteins, e.g., Liprin α3 and Rab3 (Figures S3A and S3B). We found no evidence that $Ca_V2.1$ is a SUMO-binding protein, so our hypothesis is that SUMOylation enhances RIM1α binding to $Ca_V2.1$. In agreement with this, K502R RIM1α and SENP-treated WT RIM1α bound the $Ca_V2.1$ PDZ ligand significantly less than WT RIM1α (Figures 4C and 4D). This does not represent a general modification of the availability of the PDZ domain in RIM1α by SUMOylation, given that the interaction between ELKS1b/2 and RIM1α (also via the PDZ domain) was not affected by SUMOylation (Figures S3A and S3B). Furthermore, $Ca_V2.1$-clustering defects in RIM1 KD neurons were effectively rescued by the expression of WT RIM1α, but not K502R RIM1α or a RIM1α PDZ domain-deficient construct, in which three critical PDZ domain lysines (Kaeser et al., 2011) were mutated to asparagine (3KN), (Figures 4E, 4F, S3C, and S3D). Synapsin-1 showed no differences in clustering under any of the conditions (Figures 4E and 4G). Taken together, these data demonstrate that RIM1α SUMOylation has a previously unreported role in PDZ interaction-mediated $Ca_V2.1$ clustering.

We next tested whether RIM1α SUMOylation is required for normal presynaptic Ca^{2+} signaling, using a presynaptically targeted Ca^{2+}-sensitive GFP reporter (SyGCaMP3) (Li et al., 2011). Ca^{2+} signals evoked by 40 APs at 20 Hz were significantly reduced (by ~40%) in RIM1 KD neurons, a defect that was rescued by expression of WT but not K502R RIM1α (Figures 4H and 4I). These data strongly suggest that SUMOylated RIM1α maintains normal presynaptic Ca^{2+} signaling via enhanced interactions with $Ca_V2.1$, which is required for synchronous synaptic vesicle exocytosis.

We note that our use of acute shRNA KD of RIM1 produced larger effects on activity-dependent Ca^{2+} entry and exocytosis than previous studies using knockout mice or genetic ablation in 3–5 days in vitro (DIV) neurons (e.g., Kaeser et al., 2012), in which deletion of both RIM1α and RIM2α was required to see such an effect. We perform our RIM1 KD in more mature neurons (10–11 DIV), in which active zone architecture is likely to be more established, therefore providing less scope for compensation for the loss of RIM1 by RIM2. Therefore, we believe that our approach gives a better indication of the role of the RIM1 isoform in mature synapses.

In this study, we show a previously unsuspected role for protein SUMOylation in the control of synchronous synaptic vesicle exocytosis. Specifically, we have shown a mechanism whereby SUMOylation causes the PDZ domain of RIM1α to become available for interaction with $Ca_V2.1$. This is required to promote the clustering of $Ca_V2.1$ and Ca^{2+} entry on arrival of APs at the presynaptic terminus. Although we cannot formally exclude the possibility that these presynaptic effects are due to retrograde signaling from transfected postsynaptic neurons, given the internal consistency of our different functional approaches and their close correlation with the literature, we consider this explanation unlikely. We propose that SUMOylation can act as a molecular switch in the active zone, controlling the interactions and

Figure 4. RIM1α SUMOylation Has a Critical Role in Synaptic Ca^{2+} Channel Clustering and Ca^{2+} Influx

(A) Representative blots show $Ca_V2.1$ interaction with WT and K502R RIM1α in neurons.

(B) Quantification of (A) (n = 4) is shown. Data are presented as percent (%) WT interaction levels without SENP1. ***$p < 0.001$ (one-way ANOVA). Data are represented as mean ± SEM.

(C) Representative blots show GST-$Ca_V2.1$-PDZ interaction with WT and K502R RIM1α in neurons. Pull-downs with GST-$Ca_V2.1$-PDZ were blotted for HA (RIM1α) and GST.

(D) Quantification of (C) (n = 5) is shown. Data are presented as percent (%) WT interaction levels without SENP1. *$p < 0.05$ and **$p < 0.01$ (one-way ANOVA). Data are represented as mean ± SEM.

(E) Representative images show $Ca_V2.1$ levels (green) in synapsin-1-dense regions (blue) in RIM1 KD and rescue hippocampal neurons. The scale bar represents 5 μm.

(F) Quantification of synaptic $Ca_V2.1$ in (E) (n = 12–43) is shown. ***$p < 0.001$ (one-way ANOVA) compared to both control and WT rescue. Data are represented as mean ± SEM.

(G) Quantification of synapsin-1 in (E) is shown. Data are represented as mean ± SEM.

(H) Presynaptic Ca^{2+} influx measured by SyGCaMP3 fluorescence (n = 8–10) is presented. SyGCaMP3 fluorescence is normalized to baseline and expressed as percent (%) maximum SyGCaMP3 signal obtained with 5 μM ionomycin. Panels below are representative images of SyGCaMP3 fluorescence. Data are represented as mean ± SEM. The scale bar represents 5 μm.

(I) Quantification of SyGCaMP fluorescence after 2 s of stimulation is shown. *$p < 0.05$ and **$p < 0.001$ (one-way ANOVA). Data are represented as mean ± SEM. See also Figures S2 and S3.

defining the function of different pools of multifunctional proteins, such as RIM1α. Specifically in this case, SUMOylated RIM1α is involved in the clustering of Ca^{2+} channels required for coordinated Ca^{2+} entry at the presynapse, whereas nonSUMOylated RIM1α participates in functions such as vesicle priming and docking. Such a molecular switch would help to explain how several active zone proteins participate in many diverse functions. Defining exactly how this SUMO-dependent "switching" between RIM1α-binding proteins orchestrates interactions at the active zone will provide important insight into synaptic function and dysfunction.

EXPERIMENTAL PROCEDURES

Molecular Biology

Cloning of all constructs was carried out with standard molecular biology techniques.

Biochemistry

Cultured rat cortical neurons (18 DIV) or HEK293T cells were used for SDS-PAGE, immunoblotting, immunoprecipitations, and GST pull-downs. Lysates were incubated ±20 mM NEM at 37°C for 30 min. HEK293T cell SUMOylation assays were conducted as described (Craig et al., 2012). For interactor studies, neurons infected with WT or K502R RIM1α-HA Sindbis virus were lysed on ice ±20 nM SENP1, and interactions were probed using either anti-HA immunoprecipitation or a GST-tagged $Ca_V2.1$ PDZ ligand (sequence SEDDWC).

Neuronal Cultures and Imaging

Embryonic rat hippocampal and cortical neurons were prepared as described (Martin and Henley, 2004). Neurons were typically transfected at 11 DIV and imaged 4 days later (15 DIV). Immunocytochemistry assays were performed with paraformaldehyde fixation according to standard protocols. Image and blot analysis was performed using ImageJ software, and statistical analysis was conducted using GraphPad Prism. For functional fluorescence assays, one cell with 10–13 ROIs was analyzed per repeat. Fluorescence data were first normalized to baseline and then to maximal values (F_{max}).

Fluorescent Exocytosis Assays

For FM dye experiments, hippocampal neurons were transfected with RIM1 shRNA (mCherry) and either a WT or K502R RIM1α-HA rescue construct. FM1-43 experiments were performed as described by Gaffield and Betz (2006) with 0.2 Hz imaging. SypHy and SyGCaMP3 were expressed on a pFIV RIM1 shRNA vector and cotransfected with WT or K502R mCherry-IRES-RIM1α. SypHy experiments were performed as described by Burrone et al. (2006) with imaging at 0.5, 2, or 10 Hz. SyGCaMP3 experiments used 10 Hz imaging (Li et al., 2011). Electrical field stimulation was used for all assays: 600 APs at 20 Hz to induce exocytosis of the releasable synaptic vesicle pool, 40 APs at 20 Hz for RRP release, and SyGCaMP3 experiments.

Statistical Analysis

All quantified results shown are the mean ± SEM. Statistical analyses were performed using either Microsoft Excel or GraphPad Prism. For comparison of two sets of data, one-tailed Student's t test was used. For comparison of multiple sets of data, one-way ANOVA with Bonferroni's post hoc test was used.

SUPPLEMENTAL INFORMATION

Supplemental Information includes Supplemental Experimental Procedures and three figures and can be found with this article online at http://dx.doi.org/10.1016/j.celrep.2013.10.039.

AUTHOR CONTRIBUTIONS

F.G. performed all of the biochemistry, molecular biology, and immunocytochemistry and most of the live-imaging studies and assisted in preparing the manuscript. T.J.C. performed all of the SypHy studies, developed imaging protocols, directed the project, and assisted in preparing the manuscript. D.L.R. performed the initial Ubc9 interaction screen. J.M.H. oversaw the project and prepared the manuscript. All authors discussed the results and commented on the manuscript.

ACKNOWLEDGMENTS

We thank Kai Murk for the pFIV and mCherry-IRES vectors, Phil Rubin for virus preparation, and Jon Brown for technical assistance. We also thank Ruud Toonen, Kevin Wilkinson, Keri Hildick, and Nadia Jaafari for expert experimental advice. We gratefully acknowledge Alan Morgan for pCMV RIM1α, Ruud Toonen for pCDNA3 SypHy, and Susan Voglmaier for pCDNA3 SyGCaMP3. This work was funded by the ERC, BBSRC, and MRC.

Received: July 23, 2013
Revised: October 1, 2013
Accepted: October 24, 2013
Published: November 27, 2013

REFERENCES

Burrone, J., Li, Z., and Murthy, V.N. (2006). Studying vesicle cycling in presynaptic terminals using the genetically encoded probe synaptopHluorin. Nat. Protoc. *1*, 2970–2978.

Calakos, N., Schoch, S., Südhof, T.C., and Malenka, R.C. (2004). Multiple roles for the active zone protein RIM1alpha in late stages of neurotransmitter release. Neuron *42*, 889–896.

Castillo, P.E., Schoch, S., Schmitz, F., Südhof, T.C., and Malenka, R.C. (2002). RIM1alpha is required for presynaptic long-term potentiation. Nature *415*, 327–330.

Coppola, T., Magnin-Luthi, S., Perret-Menoud, V., Gattesco, S., Schiavo, G., and Regazzi, R. (2001). Direct interaction of the Rab3 effector RIM with Ca2+ channels, SNAP-25, and synaptotagmin. J. Biol. Chem. *276*, 32756–32762.

Craig, T.J., Jaafari, N., Petrovic, M.M., Jacobs, S.C., Rubin, P.P., Mellor, J.R., and Henley, J.M. (2012). Homeostatic synaptic scaling is regulated by protein SUMOylation. J. Biol. Chem. *287*, 22781–22788.

de Jong, A.P., Schmitz, S.K., Toonen, R.F., and Verhage, M. (2012). Dendritic position is a major determinant of presynaptic strength. J. Cell Biol. *197*, 327–337.

Deng, L., Kaeser, P.S., Xu, W., and Südhof, T.C. (2011). RIM proteins activate vesicle priming by reversing autoinhibitory homodimerization of Munc13. Neuron *69*, 317–331.

Dulubova, I., Lou, X., Lu, J., Huryeva, I., Alam, A., Schneggenburger, R., Südhof, T.C., and Rizo, J. (2005). A Munc13/RIM/Rab3 tripartite complex: from priming to plasticity? EMBO J. *24*, 2839–2850.

Feligioni, M., Nishimune, A., and Henley, J.M. (2009). Protein SUMOylation modulates calcium influx and glutamate release from presynaptic terminals. Eur. J. Neurosci. *29*, 1348–1356.

Fernández-Alfonso, T., and Ryan, T.A. (2004). The kinetics of synaptic vesicle pool depletion at CNS synaptic terminals. Neuron *41*, 943–953.

Flotho, A., and Melchior, F. (2013). Sumoylation: a regulatory protein modification in health and disease. Annu. Rev. Biochem. *82*, 357–385.

Gaffield, M.A., and Betz, W.J. (2006). Imaging synaptic vesicle exocytosis and endocytosis with FM dyes. Nat. Protoc. *1*, 2916–2921.

Gundelfinger, E.D., and Fejtova, A. (2012). Molecular organization and plasticity of the cytomatrix at the active zone. Curr. Opin. Neurobiol. *22*, 423–430.

Hay, R.T. (2005). SUMO: a history of modification. Mol. Cell *18*, 1–12.

Jaafari, N., Konopacki, F.A., Owen, T.F., Kantamneni, S., Rubin, P., Craig, T.J., Wilkinson, K.A., and Henley, J.M. (2013). SUMOylation is required for glycine-induced increases in AMPA receptor surface expression (ChemLTP) in hippocampal neurons. PLoS ONE *8*, e52345.

Kaeser, P.S., Kwon, H.B., Chiu, C.Q., Deng, L., Castillo, P.E., and Südhof, T.C. (2008). RIM1alpha and RIM1beta are synthesized from distinct promoters of the RIM1 gene to mediate differential but overlapping synaptic functions. J. Neurosci. *28*, 13435–13447.

Kaeser, P.S., Deng, L., Wang, Y., Dulubova, I., Liu, X., Rizo, J., and Südhof, T.C. (2011). RIM proteins tether Ca2+ channels to presynaptic active zones via a direct PDZ-domain interaction. Cell *144*, 282–295.

Kaeser, P.S., Deng, L., Fan, M., and Südhof, T.C. (2012). RIM genes differentially contribute to organizing presynaptic release sites. Proc. Natl. Acad. Sci. USA *109*, 11830–11835.

Koushika, S.P., Richmond, J.E., Hadwiger, G., Weimer, R.M., Jorgensen, E.M., and Nonet, M.L. (2001). A post-docking role for active zone protein Rim. Nat. Neurosci. *4*, 997–1005.

Li, H., Foss, S.M., Dobryy, Y.L., Park, C.K., Hires, S.A., Shaner, N.C., Tsien, R.Y., Osborne, L.C., and Voglmaier, S.M. (2011). Concurrent imaging of synaptic vesicle recycling and calcium dynamics. Front Mol Neurosci *4*, 34.

Liu, K.S., Siebert, M., Mertel, S., Knoche, E., Wegener, S., Wichmann, C., Matkovic, T., Muhammad, K., Depner, H., Mettke, C., et al. (2011). RIM-binding protein, a central part of the active zone, is essential for neurotransmitter release. Science *334*, 1565–1569.

Lonart, G. (2002). RIM1: an edge for presynaptic plasticity. Trends Neurosci. *25*, 329–332.

Martin, S., and Henley, J.M. (2004). Activity-dependent endocytic sorting of kainate receptors to recycling or degradation pathways. EMBO J. *23*, 4749–4759.

Martin, S., Nishimune, A., Mellor, J.R., and Henley, J.M. (2007). SUMOylation regulates kainate-receptor-mediated synaptic transmission. Nature *447*, 321–325.

Müller, M., Liu, K.S., Sigrist, S.J., and Davis, G.W. (2012). RIM controls homeostatic plasticity through modulation of the readily-releasable vesicle pool. J. Neurosci. *32*, 16574–16585.

Schoch, S., Castillo, P.E., Jo, T., Mukherjee, K., Geppert, M., Wang, Y., Schmitz, F., Malenka, R.C., and Südhof, T.C. (2002). RIM1alpha forms a protein scaffold for regulating neurotransmitter release at the active zone. Nature *415*, 321–326.

Schoch, S., Mittelstaedt, T., Kaeser, P.S., Padgett, D., Feldmann, N., Chevaleyre, V., Castillo, P.E., Hammer, R.E., Han, W., Schmitz, F., et al. (2006). Redundant functions of RIM1alpha and RIM2alpha in Ca(2+)-triggered neurotransmitter release. EMBO J. *25*, 5852–5863.

Shalizi, A., Gaudillière, B., Yuan, Z., Stegmüller, J., Shirogane, T., Ge, Q., Tan, Y., Schulman, B., Harper, J.W., and Bonni, A. (2006). A calcium-regulated MEF2 sumoylation switch controls postsynaptic differentiation. Science *311*, 1012–1017.

Südhof, T.C., and Rizo, J. (2011). Synaptic vesicle exocytosis. Cold Spring Harb. Perspect. Biol. *3*, a005637.

Wang, Y., Okamoto, M., Schmitz, F., Hofmann, K., and Südhof, T.C. (1997). Rim is a putative Rab3 effector in regulating synaptic-vesicle fusion. Nature *388*, 593–598.

Supplemental Experimental Procedures

Molecular Biology: Cloning of all constructs was carried out using standard molecular biology methods. For SypHy and SyGCaMP3 experiments, SypHy or SyGCaMP3 were expressed on a pFIV plasmid which also expressed RIM1 shRNA (vector made in house, shRNA target sequence **gaggaggagaggaacattat** of *Rattus Norvegicus* RIM1α). RIM1α rescue constructs (carrying silent point mutations which made RIM1α insensitive to shRNA) were expressed either in a custom made mCherry-IRES-RIM1α vector or modified from pCMV-HA-RIM1α. FM dye experiments used a pFIV vector expressing RIM1 shRNA and mCherry. For all knockdown studies, 'Control' cells were transfected with pFIV (expressing mCherry, SypHy or SyGCaMP3) without shRNA. GST-tagged $Ca_V2.1$ PDZ ligand was produced by cloning synthetic DNA oligonucleotides into pGEX4T-1 vector. All subcloning and mutagenesis reactions were performed according to standard protocols. Sindbis viruses were produced from pSinRep5 vector as described previously (Craig et al., 2012).
Biochemistry: For all immunoprecipitations, antibodies were bound to Protein G sepharose. SUMO co-immunoprecipitation was performed from cultured rat cortical neurones (Martin et al., 2007) (DIV 18) using 1 µg of anti SUMO D11 antibody (Santa Cruz). Cells were lysed on ice (lysis buffer: 150 mM NaCl, 25 mM HEPES, 1% Triton and 0.1% SDS – pH 7.4) and incubated at 37°C for 30 minutes with or without 20 nM NEM before immunoprecipitation. Western blotting was performed using anti-RIM1/2 antibody (Synaptic Systems). For the HEK293T SUMOylation assay (Craig et al., 2012), cells were transfected with RIM1α-HA, Ubc9-Flag and either active SUMO-1-GG-YFP or non-conjugatable SUMO-ΔGG-YFP for 48 hours before lysis on ice in buffer containing 20 nM NEM. HA co-immunoprecipitation was performed using 2 µg mouse anti-HA antibody (Sigma) and anti-GFP (Roche). For interactor studies, RIM1α co-immunoprecipitation was performed from neurones infected with Sindbis virus encoding WT or K502R RIM1α-HA for 18 hours using 2 µg of mouse/rabbit anti-HA antibody (Sigma). For Rab3 interactions, immunoprecipitations were carried out in the presence of 0.5 mM GTPγS. Samples were lysed on ice in buffer with or without 20 nM SENP1 and blotted for the protein of interest. Free glutathione-S-transferase (GST) and the $Ca_V2.1$ PDZ ligand plus two preceding amino acids (sequence SEDDWC) fused to GST were expressed in BL21 (DE3) *E.Coli* and purified on glutathione sepharose beads. Affinity pulldowns were performed from neuronal lysate infected with WT or K502R RIM1α-HA (lysis/binding buffer: 300 mM NaCl, 25 mM HEPES, 2 mM DTT and 1% Triton – pH 7.4. Wash buffer in mM: 150 mM NaCl, 25 mM HEPES, 1 mM DTT and 1% Triton – pH 7.4).
Antibodies: Other antibodies used were anti-RIM1 (BD Biosciences), anti-βIII tubulin (Sigma Aldrich), mouse/rabbit anti-HA (Sigma Aldrich), anti-GST (Amersham), anti-GFP (Roche), mouse anti-Synapsin1 (BD Biosciences), rabbit anti-Synapsin1 (Novus Biologicals), anti-Munc13 (BD Biosciences), anti-Rab3 (BD Biosciences), anti-Liprin α3 (Synaptic Systems), anti-ERC1b/2 (ELKS1b/2) (Synaptic Systems), anti-$Ca_V2.1$ (Alomone Labs) and anti-SUMO-1 21C7 (Developmental Studies Hybridoma Bank, Iowa).
Immunocytochemistry: Immunocytochemistry assays were performed with paraformaldehyde fixation according to standard protocols. Cells were permeabilized in 0.1% Triton except for SUMO-1 staining, in which 20 µg/ml digitonin was used in order to avoid nuclear permeabilisation and thereby reduce the intense SUMO-1 staining normally seen in cell nuclei. All quantification was performed using ImageJ software. In all cases, n = mean of 10-12 ROIs taken from one cell. At least three independent experiments (i.e. on different neuronal cultures on different days) were performed in all cases, with all results normalised to the average of the control values (set to 100%) for each batch of cells. For colocalisation experiments, Mander's test of colocalisation was used. For Fig. 1D, the Mander's M1 value recorded represents the overlap of SUMO-1 signal with that of RIM1/2. For Supplemental data Fig. 1E-F, the Mander's M1 value represents the overlap of HA (RIM1α) signal with that of Synapsin-1 and values are normalised to the mean WT RIM1α colocalisation index. For $Ca_V2.1$ clustering experiments, synaptic $Ca_V2.1$ was identified by picking ROIs from Synapsin-1 dense regions (as in (Kaeser et al., 2011)).

Cell Culture: Embryonic cortical and hippocampal neurones were isolated and maintained as previously described (Martin and Henley, 2004). For all biochemistry, cortical neurones were used at DIV18. For all live cell imaging, hippocampal neurones were used at DIV15. For FM-dye experiments, hippocampal neurons were typically transfected at DIV 11 with RIM1 shRNA (mCherry) and either WT or non-SUMOylatable (K502R) RIM1α-HA rescue and imaging experiments were performed four days later. For SypHy and SyGCaMP3 experiments, transfections were performed in a similar manner, but with SypHy or SyGCaMP3 expression on the shRNA vector and a mCherry-IRES-RIM1α rescue construct. All neuronal transfections were performed using Lipofectamine 2000 (Invitrogen) according to manufacturer's instructions.

FM1-43 Experiments: FM1-43 dye loading and unloading was carried out as described (Gaffield and Betz, 2006). Cells were kept in HBS (in mM: 140 NaCl, 5 KCl, 1.8 $CaCl_2$, 0.8 $MgCl_2$, 25 HEPES and 0.9 g/litre glucose - pH 7.4) with 25 µM CNQX and 50 µM D-AP5 throughout. The entire releasable pool of vesicles was FM labelled by stimulating with 45 mM KCl for 90 seconds in the presence of dye, followed by an additional 90 seconds of dye exposure. Following 10 minutes of washing in dye-free solution, cells were imaged. Images of FM1-43 fluorescence were taken every 5 seconds by confocal microscopy. Baseline fluorescence was captured for 60 seconds before a stimulation of 600 action potentials (APs) at 20 Hz was applied to induce bulk exocytosis and thus dye unloading. After 2 minutes, the nerve terminal was stimulated with 900 APs at 30 Hz to enable complete unloading of all releasable FM dye (to reveal terminal background). For each cell, 12 ROIs were analysed where each ROI encapsulated a single synaptic bouton. Values were normalised to terminal background. Slope analysis was performed on the initial 15 seconds of stimulation (during which all profiles were linear) by linear regression.

Synaptophysin-pHluorin (SypHy) Experiments: SypHy experiments were performed as described (Burrone et al., 2006). Exocytosis was triggered using electrical field stimulation, using 1 ms pulses (APs) of either 20 mA or 50 V (which were found to yield identical results). RRP stimulation was triggered by 40 APs at 20 Hz. Fusion of all releasable synaptic vesicles (reserve pool or RP)_was triggered by 600 APs at 20 Hz. All experiments were performed in HBS with 50 µM D-AP5, 25 µM CNQX and 1 µM bafilomycin A. Perfusion with NH_4Cl (pH 7.4), was used to reveal total levels of SypHy loading of neurones at the end of each experiment. Images were taken at 0.5,2 or 10 Hz depending on the experiment, using a CCD camera with a GFP filter cube. For each cell, 13 ROIs were analysed, with non-responsive ROIs discounted. Data were first normalised to background levels (i.e. $\Delta F/F_0$), and then expressed as a percentage of fluorescence after NH_4Cl perfusion (F_{max}). Slope analysis was performed by linear regression on the initial 10 seconds of stimulation (during which all profiles were linear) for RP release, and for the full 2 seconds of stimulation for RRP release.

SyGCaMP3 Experiments: All experiments were performed in HBS with 25 µM CNQX and 50 µM D-AP5. As with SyPhy experiments, RRP stimulation was triggered by 40 APs at 20 Hz after steady baseline recordings were obtained. Images were taken at 10 Hz using a CCD camera with a GFP filter cube. Maximal SyGCaMP3 signal was obtained by 5 minute incubation with 5 µM ionomycin made up in HBS. Data were first normalised to background levels (i.e. $\Delta F/F_0$) and then expressed as a percentage of fluorescence after ionomycin perfusion (F_{max}). For all functional assays, data is collated from at least 3 individual experiments where n=one cell and for each cell, 10-13 ROIs were analysed. Image analysis was performed using ImageJ software as described (Gaffield and Betz, 2006).

Statistical Analysis: Graphpad Prism software (Graphpad Inc.) was used for graph plotting and statistical analysis. For comparison of multiple data sets, 1-way ANOVA with Bonferroni's post-hoc test was used. For comparison of 2 data sets, a 1-tailed Student's *t*-test was used. Curve fitting for Fig. 3C was performed using Graphpad Prism. Control and WT rescue traces were fitted with a Boltzman Sigmoidal curve whereas KD and K502R rescue traces were fitted with linear regression. All data is reported as mean ± SEM.

Supplemental Figure Titles and Legends

Figure S1 – A501S RIM1α is non-SUMOylatable, Related to Figure 1

A: Confirmation that lysine 502 is the only SUMOylation site on RIM1α. WT and A501S RIM1α were used in the SUMOylation assay in HEK293T cells (Fig. 1F,G). Western blot for HA (RIM1α).

Figure S2– RIM1 knockdown-rescue, equal expression and normal targeting/interaction of K502R RIM1α, and FM1-42 uptake in KD-A501S rescue cells, Related to Figures 2, 3 and 4.

A: Representative blot showing knockdown of exogenously expressed RIM1α-HA and rescue with both WT and non-SUMOylatable K502R RIM1α in HEK293T cells. β-tubulin is shown as a loading control.

B: Quantification of (A). RIM1α expression levels are expressed as % control cells not transfected with RIM1 shRNA. (n=4) ***p<0.001 (1-way ANOVA). Data are represented as mean ± SEM.

C: Representative images for hippocampal neurones stained for RIM1/2 (green) after cotransfection with RIM1 shRNA (mCherry, red) and either WT or K502R RIM1α rescue (green). Scale bar - 10 µm.

D: Quantification of (C). RIM1α expression levels are expressed as % control cells not transfected with RIM1 shRNA. (n=10-16) ***p<0.001 (1-way ANOVA). Data are represented as mean ± SEM.

E: Representative images for RIM1α-HA colocalisation with Synapsin-1. Hippocampal neurones infected with Sindbis virus expressing either WT or K502R RIM1α-HA stained 18 hours after infection for presynaptic protein Synapsin-1 (red) and HA (green). Arrows highlight regions of colocalisation. Scale bar - 5 µm.

F: Quantification of (E). Mander's M1 coefficient for colocalisation of HA (RIM1α) with Synapsin-1 (n=6, 6 ROIs per cell). Data are expressed as % of the M1 coefficient of the WT condition. Data are represented as mean ± SEM.

G: Representative images for FM1-43 dye uptake by high K^+ (45 mM) stimulation in hippocampal neurones. Scale bar - 5 µm.

H: Quantification of (B). FM1-43 uptake for control, RIM1 KD, WT rescue, K502R rescue and A501S rescue conditions. Normalised fluorescence refers to average FM1-43 pixel intensity as % mean fluorescence of control neurones. (n=5-8) ***p<0.001 (1-way ANOVA). Data are represented as mean ± SEM.

Figure S3 – RIM1α interaction with Liprin α3 and Rab3 is not SUMO-dependent and 3KN RIM1α does not bind $Ca_V2.1$, Related to Figure 4.

A: Representative Rab3, Liprin α3 and ELKS1b/2 blots of HA (RIM1α) immunoprecipitation from cortical neurones virally infected with WT or K502R RIM1α-HA.

B: Quantification of (A) (n=3-4). Data are represented as mean ± SEM.

C: Representative blots showing GST-$Ca_V2.1$-PDZ interaction with WT and 3KN RIM1α in HEK293T cells. Pulldowns with GST-$Ca_V2.1$-PDZ were blotted for HA (RIM1α) and GST.

D: Quantification of (C) (n=3). Data are presented as % WT interaction. ***p<0.001 (1 way ANOVA). Data are represented as mean ± SEM.

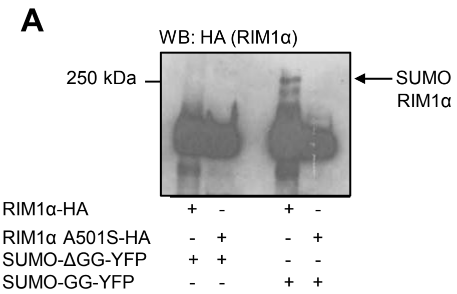

Girach et al. – **Figure S1**

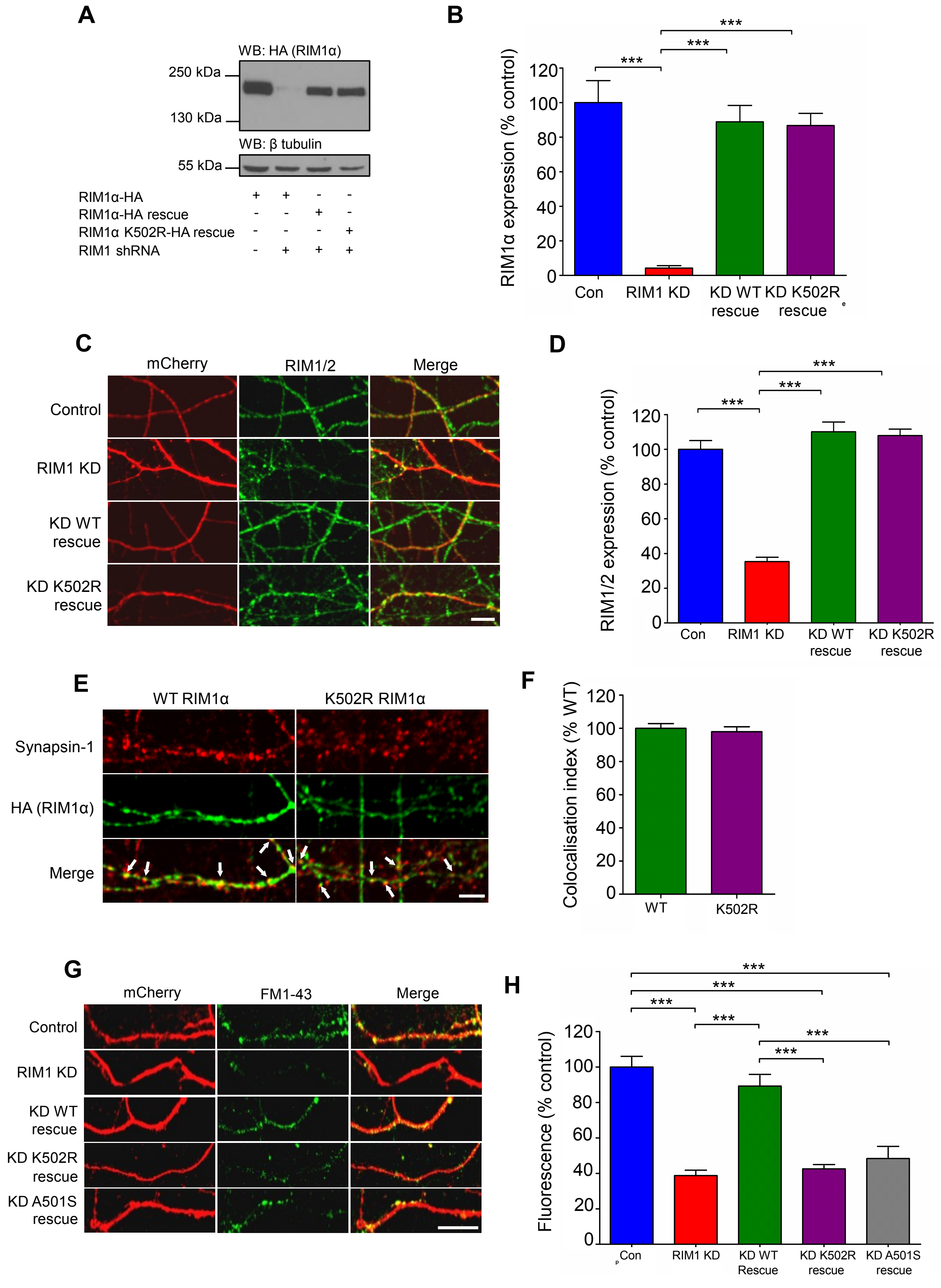

Girach et al. – **Figure S2**

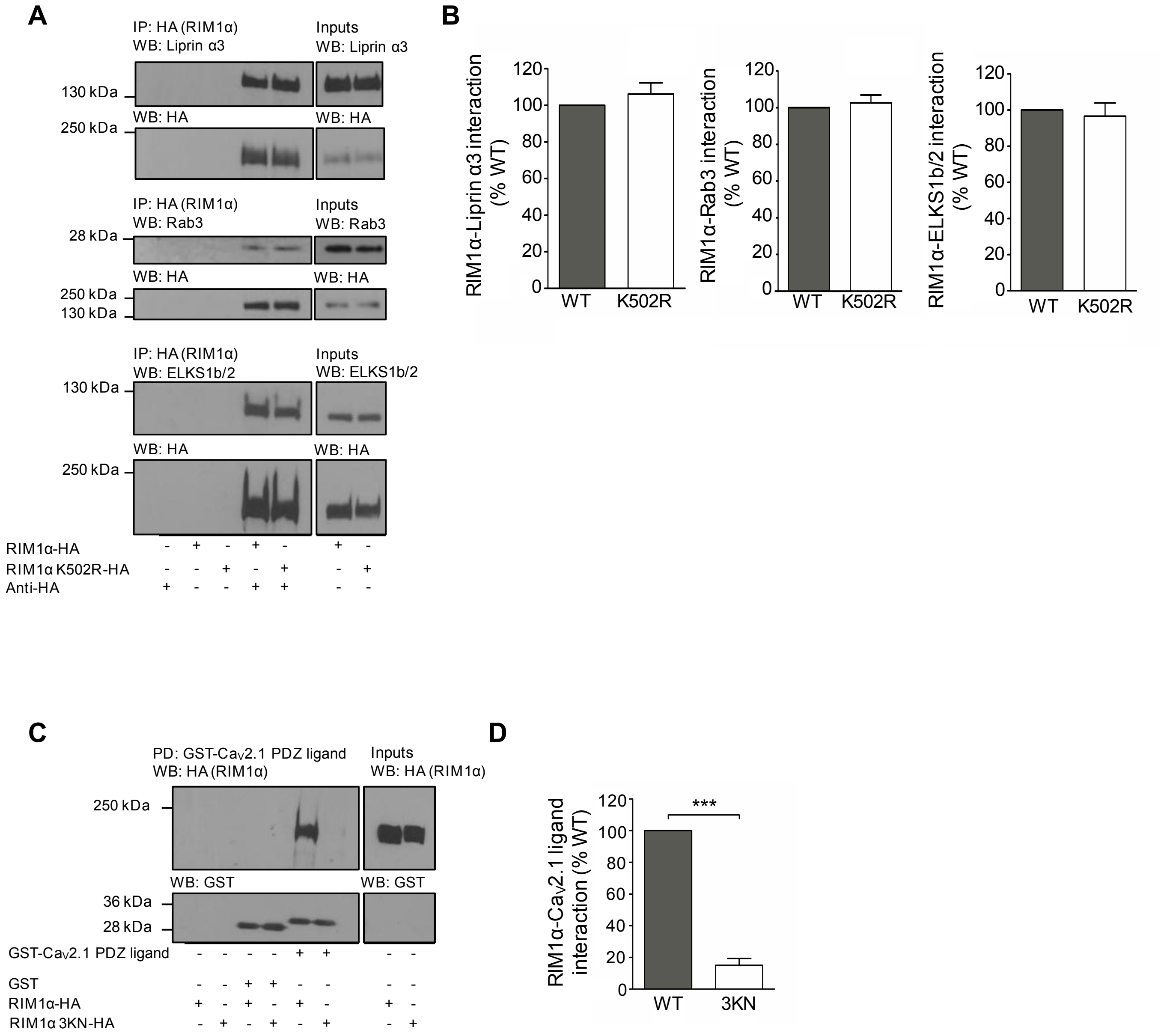

Girach et al. – **Figure S3**
